# Divergence in sex peptide-mediated female post-mating responses in *Drosophila melanogaster*

**DOI:** 10.1098/rspb.2018.1563

**Published:** 2018-09-12

**Authors:** Kristina U. Wensing, Claudia Fricke

**Affiliations:** 1 Institute for Evolution and Biodiversity, University of Muenster, Muenster 48149, Germany; 2 Muenster Graduate School of Evolution, University of Muenster, Muenster 48149, Germany

**Keywords:** sexual conflict, cost of mating, sexually antagonistic coevolution, sex peptide, post-mating response

## Abstract

Transfer and receipt of seminal fluid proteins crucially affect reproductive processes in animals. Evolution in these male ejaculatory proteins is explained with post-mating sexual selection, but we lack a good understanding of the evolution of female post-mating responses (PMRs) to these proteins. Some of these proteins are expected to mediate sexually antagonistic coevolution generating the expectation that females evolve resistance. One candidate in *Drosophila melanogaster* is the sex peptide (SP) which confers cost of mating in females. In this paper, we compared female SP-induced PMRs across three *D. melanogaster* wild-type populations after mating with SP-lacking versus control males including fitness measures. Surprisingly, we did not find any evidence for SP-mediated fitness costs in any of the populations. However, female lifetime reproductive success and lifespan were differently affected by SP receipt indicating that female PMRs diverged among populations. Injection of synthetic SP into virgin females further supported these findings and suggests that females from different populations require different amounts of SP to effectively initiate PMRs. Molecular analyses of the SP receptor suggest that genetic differences might explain the observed phenotypical divergence. We discuss the evolutionary processes that might have caused this divergence in female PMRs.

## 1. Introduction

Mating induces dramatic changes in female behaviour, physiology and gene expression that together constitute female post-mating responses (PMRs). In insects, two striking PMRs are increased egg production and a decreased receptivity towards courting males [1]. The act of mating, but particularly the receipt of sperm and proteins as part of the seminal fluid, induces female PMRs [2]. Male seminal fluid proteins (SFPs) are found in many taxa [2,3] and have been particularly well studied in *Drosophila melanogaster* where specific functions for several of the more than 130 SFPs have been identified and are responsible for invoking the majority of the female PMRs [4]. These SFPs are known to evolve rapidly [5,6] and coevolve with targets inside the female [7]. While male SFPs, their function and evolution have been intensively studied, the evolution of variance in female PMRs remains understudied.

In *D. melanogaster,* the transfer of SFPs benefits male reproductive success [1] and allows females to coordinate reproductive processes with the receipt of sperm as well as aid sperm management [4]. Despite these benefits, females suffer reduced longevity and lifetime reproductive success (LRS) from repeated receipt of SFPs [8] as part of the cost of mating. Therefore, *Drosophila* SFPs have been proposed as mediators of sexual conflict and targets of sexually antagonistic coevolution [8–10].

Sexual conflict can arise when the sexes follow different routes to maximize fitness, creating the potential for sexually antagonistic coevolution [11]. Theory predicts that the evolution of a trait that increases reproductive success in one sex while depressing the fitness of the other leads to coevolutionary cycles of adaptation and counteradaptation between the sexes [11,12]. This typically generates male persistence and female resistance traits. The latter evolve in response to male persistence traits to reduce the fitness costs imposed on females by reproductive interactions [11]. Hence, the evolution of female PMRs mediated by SFPs may be shaped by sexual conflict and sexually antagonistic coevolution.

Most empirical studies have focused on demonstrating and understanding the nature of male persistence traits but data on female resistance are scarce [11,13,14], probably because female traits are notoriously difficult to study. Notable exceptions include studies of morphological male antagonistic and corresponding female resistance traits which affect the mating rate, like the male grasping and female antigrasping traits in *Gerris* spp waterstriders [15,16] and diving beetles of the Dytiscidae family [17,18] (for a review, see [14]). Interestingly, the shape of the antigrasping traits in *Gerris incognitus* was moulded simultaneously by male–female coevolution and ecological factors [19]. Furthermore, the expression of female costs of mating in *D. melanogaster* is condition-dependent, e.g. on variation in available diet [20], demography [21,22] or the physical environment [23]. This can shift the economics of mating potentially altering coevolutionary dynamics [13,19,24]. Hence, the interplay between ecology and sexually antagonistic coevolution might shape the evolution of intraspecific variation in male and female reproductive traits.

In contrast to morphological traits, while we have started to identify the physiological and neuronal pathways and players mediating female PMRs in response to male chemical sexually antagonistic traits such as SFPs, we lack an understanding of how they coevolve with male traits [14]. Intriguingly, so far the female receptor for only one male SFP has been identified. The sex peptide receptor (SPR) is required for the PMRs mediated by receipt of male sex peptide (SP) [25]. Single nucleotide polymorphisms in the *SP* and *SPR* genes and their interaction affect sperm competition success and female remating [26]. This makes them promising candidates to study sexually antagonistic coevolution at the phenotypic and molecular level. The 36-amino acid long SP induces oviposition and decreases a female's willingness to remate [27], which directly benefits the male [28,29] and affects sperm competition outcomes [28,30]. Additionally, receipt of SP is one major contributor to the costs of mating in female *D. melanogaster* [10]. The opposing fitness consequences of SP transfer and receipt for, respectively, males and females, are expected to mediate sexual conflict over reproductive decisions and investment [10,28,31].

In this study, we investigated whether female PMRs to a specific sexually antagonistic signal, ejaculatory SP, diverged across three wild-type *D. melanogaster* populations, and whether we can detect signs of sexually antagonistic coevolution between SP and female PMRs. We combined tests for phenotypic variation in female PMRs with an investigation of expression levels of and genotypic variation in the *SPR* gene which is crucial for invoking the PMRs.

## 2. Methods

### (a) Fly stocks and culturing methods

We used flies from three different *D. melanogaster* wild-type populations: Dahomey, Innisfail and Melbourne. All strains were kept in the laboratory at 25°C and 60% humidity on a 12 L : 12 D cycle and provided with standard sugar-yeast (SY) medium [28]. Dahomey was collected in Africa in the 1970s, while the Innisfail and Melbourne populations were collected in 2008 from the tropical and temperate ends, respectively, of a transect along the eastern coast of Australia [32]. We used SP knockout and appropriate control males [27] to test female responses to SP receipt. Males lacking SP (*SP*^0^) and genetically matched SP producing control males (*SP^+^*) were in the Dahomey genetic background and generated as described in [33].

Standardized females (see below) were from a randomly chosen isofemale (i.e. inbred) line of the Drosophila Genetic Reference Panel (line ID 776) [34]. For further details on fly stock maintenance, see electronic supplementary material.

To obtain experimental adults, the parental generation was allowed to oviposit on agar-grape juice plates supplemented with fresh yeast paste. Larvae were collected the following day at a density of 100 per vial containing 7 ml of SY medium supplemented with live yeast. We separated adults directly after eclosion to ensure virginity and held them in single sex groups of 20 individuals per vial until the start of the experiment.

All experiments were carried out at 25°C and 60% humidity in glass vials supplemented with 7 ml SY food and live yeast granules or paste.

### (b) Single mating experiments

We carried out three separate experiments in which we measured female egg laying and remating behaviour 24 h after a single mating. In the first, we mated females of the three populations to their own males to measure the expression of female PMRs when mated to coevolved males. In the second, we mated males from the three populations to standardized females to compare the males' abilities to induce a PMR. While in the third, we mated females from the three populations to an *SP*^0^ or *SP^+^* male to determine whether there were population differences in the strength of their SP-mediated PMRs. In all three experiments, we set up 40 pairs per treatment; for details on the experimental procedure, see electronic supplementary material.

### (c) Costs of mating incurred by wild-type females continuously exposed to *SP*^0^ and *SP*^+^ males

Here, we tested the effects of continuous exposure to *SP*^0^ or *SP^+^* males on LRS and lifespan in Dahomey, Innisfail and Melbourne females. We kept groups of three females continuously with three males of either genotype until their natural death. A total of 210 females (24–48 h post-eclosion) from each population were split evenly across the two male genotypes (i.e. 35 vials per population per male genotype). Males were 4 days post-eclosion to ensure sexual maturity [33]. Female survival was checked daily. We further recorded mating activity and the number of offspring produced twice a week. Mating activity was recorded for 3 h directly after lights on by scoring the number of mating pairs every 20 min. After the end of the spot check period, 21 randomly selected females per treatment were placed individually into fresh vials and allowed to oviposit for 18 h. The next morning, we moved females back into mating groups. We counted the number of eggs produced per female and incubated vials for 12 days and counted the number of eclosing adult offspring. For each treatment group (female population × male genotype), we stopped the assay when less than 21 females remained. In total, we measured six egg-laying time points for Melbourne females, five for Dahomey and Innisfail females mated to *SP^+^* males, and four time points for Innisfail females mated to *SP*^0^ males. We totalled the number of eggs and adult offspring produced across all measured time points as a proxy of LRS.

Throughout the experiment, we maintained groups by transferring them to fresh food vials every 2 days using CO_2_ anaesthesia. At these transfers, we combined females across vials when dead females were found, to keep density constant. In addition, dead males were replaced at this opportunity when necessary. Once a week males were replaced with new 4-day-old males of the appropriate genotype to avoid male age as a confounding factor. At the same time, females were randomly mixed across vials within treatment to minimize the effects of mating group constellation.

### (d) Injection of synthetic SP into virgin females

We injected different concentrations of synthetically derived SP (*n* = 21) into the abdomens of virgin 4-day-old females from the three populations to determine whether females differ in the amount of SP needed to display PMRs. Six hours after the injection, we measured the proportion of injected females (i) laying eggs (as not all females laid eggs) and (ii) mating with a 4-day-old Dahomey male. For details of the experimental procedure, see electronic supplementary material.

### (e) SPR expression and SP and SPR haplotype networks

We used quantitative Real-Time PCR (qPCR) to measure relative expression levels of *SPR* in the female abdomen to quantify *SPR* expression in the reproductive tract [25]. RNA isolation from female abdomen, cDNA synthesis, qPCR and relative expression calculation were carried out as described in [35]. For detailed methods and primer sequences, see electronic supplementary material.

We sequenced the whole *SP* gene (FlyBase ID: FBgn0003034) and exon 6 of the *SPR* gene (FBgn0029768) in all three wild-type populations. A previous study identified 16 SNPs within exon 6 of the SPR of which nine had significant effects on egg laying, remating behaviour or egg hatchability [26]. We used the sequence information to build haplotype networks using statistical parsimony implemented in the TCS software [36]. To do this, we first isolated genomic DNA from single flies and amplified *SP* and exon 6 of *SPR* in a standard PCR with gene-specific primers (see electronic supplementary material). PCR products were then purified with alkaline phosphatase and exonuclease I (both from Thermo Scientific); correct product sizes were verified on 1%-agarose gels and then sent to GATC Biotech AG (Germany) for Sanger sequencing with the primers used for amplification. The resulting sequences were manually edited and aligned using PhyDE v. 0.997. In total, we used sequences from 14 Dahomey, 16 Innisfail and 10 Melbourne individuals for the SP network and six Dahomey, four Innisfail and five Melbourne individuals to build the SPR exon 6 network with the connection limit among haplotypes set to 97% and gaps treated as missing data.

### (f) Statistical analysis

All statistical analyses were performed in RStudio v. 0.99.467 [37] using R v. 3.4.0 [38]. We specified generalized linear models (GLMs) [39] with the appropriate data distributions. Specifics on data distributions and use of the quasi-extension to account for overdispersion are given with the results. We tested for treatment effects using likelihood ratio tests by dropping terms. Post hoc tests for pair-wise comparisons were done using the *glht* function from the ‘multcomp’ package v. 1.4-8 (for main terms) [40] and the *testInteractions* function from the ‘phia’ package v. 0.2-1 (for interaction effects) [41]. Both tests use the Holm method to adjust *p*-values for multiple testing [42]. We performed the non-parametric Scheirer–Ray–Hare test [43] in cases where appropriate GLMs could not be fitted.

To analyse the mating data from the spot checks in the continuous exposure experiment, we calculated the total number of mating opportunities (sum of females alive for all days when checks were done) and summed the number of matings observed over all spot checks and used these sums in a *χ*^2^-test of association. We used the *chisq.post.hoc* function from the ‘fifer’ package v. 1.1 [44] to do pair-wise comparisons of the populations.

Graphs were made using the ‘gplots’ package v. 3.0.1. [45]. Unless stated otherwise, we present means ± s.e. calculated from raw data. For proportion data, we calculated the standard error as 
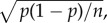
 where *p* is the proportion across all replicates and *n* is the sample size.

## 3. Results

### (a) Single mating experiments

#### (i) Population divergence in female PMRs

When mating once with their coevolved males, Innisfail females laid most eggs within the 24 h following mating, while Dahomey females produced the least eggs (GLM with quasi-poisson data distribution: *F*_2*,*110_ = 58.03, *p* < 0.001, electronic supplementary material, figure S1a). In addition, Innisfail females remated significantly more often than Dahomey females but not Melbourne females (GLM with binomial data distribution: 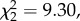 *p* = 0.01, electronic supplementary material, figure S1b). Hence, all three populations differed significantly in their coevolved PMRs for these two traits and, in the following, we picked apart male from female contributions to these responses.

#### (ii) Wild-type male ability to elicit female PMRs

Male population origin had no significant effect on egg-laying rate after a single mating to the standard isoline females (GLM with quasi-poisson data distribution: *F*_2*,*99_ = 3.05, *p* = 0.052; electronic supplementary material, figure S2a). Similarly, there was no difference in the proportion of females remating (GLM with binomial data distribution: 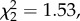 *p* = 0.466; electronic supplementary material, figure S2b). Hence, overall males from the three populations were similar in their ability to elicit SP-dependent female PMRs for this particular isoline.

#### (iii) Response to receipt of sex peptide in wild-type females after a single mating

**Figure 1.**
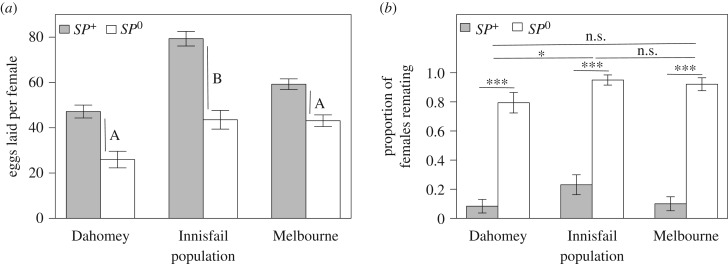
Female PMRs to receipt of SP 24 h after a single mating to either an SP*^+^* (grey bars) or an *SP*^0^ (white bars) male. (*a*) Mean number of eggs (±s.e.) produced per female. Different letters correspond to significant differences (*p* < 0.05) in contrasts of female population × male genotype interactions. (*b*) Proportion of females remating (± s.e.) with a Dahomey male within 1 h. Asterisks correspond to significant differences (***p < 0.001, **p < 0.01, *p < 0.05, n.s. = non-significant) in remating propensity depending on male genotype within populations and between populations.

SP receipt had the expected effects on females in all three populations: females first mated to *SP^+^* males laid more eggs than females mated to *SP*^0^ males. However, this egg boost was significantly stronger in Innisfail females than in Dahomey and Melbourne females as indicated by the significant male genotype × female population interaction (figure 1*a* and table 1). As expected, females first mated to *SP^+^* males also had a reduced propensity to remate (*SP^+^*: 13.9 ± 3.2%, *SP*^0^: 89.3 ± 2.9%) with the extent of this reduction not significantly different among populations (figure 1*b* and table 1). However, across populations females overall differed significantly in their willingness to remate (table 1), with Innisfail females having a higher remating propensity (59.5 ± 5.5%) than Dahomey females (42.3 ± 5.9%). Melbourne females were intermediate, not differing significantly from either of the other two populations (50.0 ± 5.7%). The proportion of eggs that developed into adults (on average 86.1 ± 2.3%) was not affected by male genotype (Scheirer–Ray–Hare test, d.f. = 1, *p* = 0.27), female population (d.f. = 2, *p* = 0.97), nor their interaction (d.f. = 2, *p* = 0.70).

**Table 1.** Test statistics of GLMs testing for the effect of male genotype (*SP*^0^/*SP^+^*), female population and their interaction on the number of eggs laid by a once-mated female and on female remating behaviour 24 h after a first mating.

factor/term	deviance	d.f.	test statistic	*p*-value
*number of eggs (Gaussian data distribution*)			*F*	
female population	115 791	2	29.10	<0.001
male genotype	126 723	1	85.10	<0.001
female population × male genotype	92 178	2	5.25	0.006
*remating behaviour (binomial data distribution)*			*χ*^2^	
female population	169.95	2	8.11	0.017
male genotype	310.39	1	149.45	<0.001
female population × male genotype	160.94	2	0.70	0.71

### (b) Cost of mating response of wild-type females continuously exposed to *SP*^0^ and *SP*^+^ males

**Figure 2.**
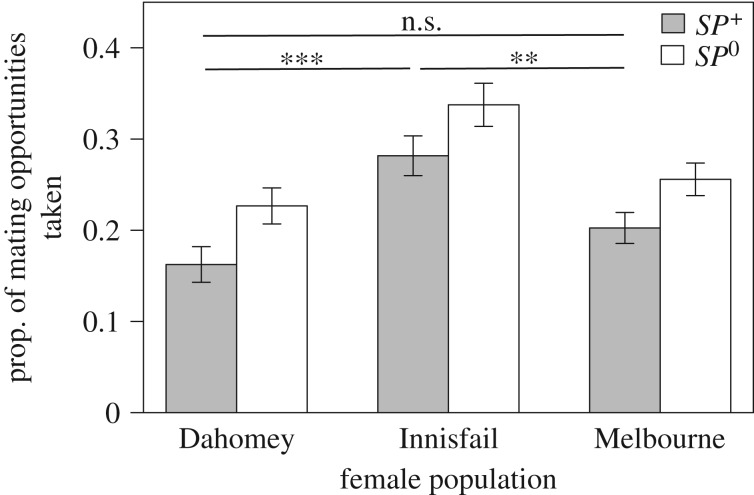
Mating frequency of females continuously exposed to *SP^+^* (grey bars) or *SP*^0^ (white bars) males throughout their lives. Mating frequency was recorded twice a week for 3 h directly after lights on by checking the number of mating pairs every 20 min until all females were dead. Asterisks correspond to significant contrasts (***p < 0.001, **p < 0.01, *p < 0.05, n.s. = non-significant) in mating frequency between females from different populations mated to *SP^+^* males (the pattern was similar when comparing females from different populations mated to *SP*^0^ males).

In general, females held with *SP*^0^ males mated significantly more often (Pearson's *χ*^2^-test: 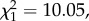 *p* = 0.002, figure 2). When comparing the three wild-types exposed to *SP^+^* males separately, we found significant differences with Innisfail females showing the highest mating frequency (*χ*^2^-test: 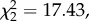 *p* < 0.001, figure 2).

**Figure 3.**
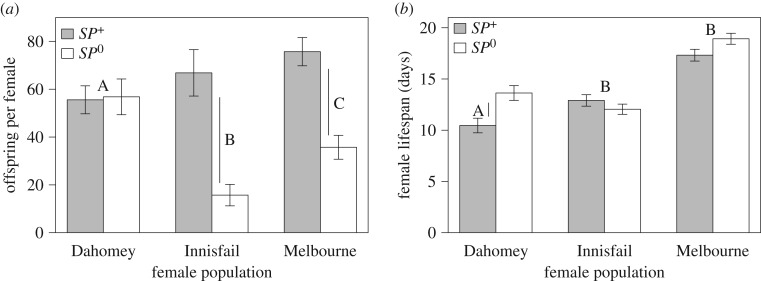
PMRs after continuous exposure to *SP^+^* (grey bars) and *SP*^0^ (white bars) males in Dahomey, Innisfail and Melbourne females. (*a*) LRS was measured as mean number of adult offspring (±s.e.) per female. (*b*) Mean female lifespan (±s.e.) in days. Different letters indicate significant contrasts (*p* < 0.05) for female population × male genotype interactions on LRS and lifespan.

We estimated female LRS as the total number of adult offspring developed from vials in which 21 randomly chosen individualized females oviposited twice a week for 18 h. The LRS estimates were significantly affected by the male genotype × female population interaction (GLM with quasi-poisson distribution: *F*_2,120_ = 11.38, *p* < 0.001). Both Innisfail and Melbourne females had higher LRS estimates when exposed to *SP^+^* males, but Innisfail females boosted offspring production more strongly when receiving SP, while Melbourne females still produced nearly half as many offspring even when not receiving SP. By contrast, Dahomey females produced similar numbers of offspring irrespective of male genotype (figure 3*a*).

Female lifespan was not only affected by their population of origin, but also whether males transferred SP or not (GLM with Gamma data distribution: *F*_2*,*615_ = 9.27, *p* = 0.006). For both the Dahomey and Melbourne populations, females continuously exposed to *SP^+^* males had a shorter lifespan than females exposed to *SP*^0^ males, while in the Innisfail population, this pattern reversed and females exposed to *SP^+^* males lived on average longer than females exposed to *SP*^0^ males. However, only Dahomey and Innisfail females differed significantly from each other in the modulation of their lifespan in response to repeatedly receiving SP versus not at all (figure 3*b*).

We used the LRS estimates and lifespan data to calculate the intrinsic population growth rate *r* as an index of fitness with similar results (electronic supplementary material, figure S3).

### (c) Injection of synthetic SP into virgin females

We first tried injecting synthetic SP at concentrations between 0 and 5 pmol as used in [46] in a few females (less than or equal to 10) but found that Innisfail and Melbourne females did not display PMRs at these concentrations (electronic supplementary material, figure S4). We therefore used higher concentrations ranging from 5 to 10 pmol.

The proportion of females that laid eggs within 6 h was not significantly affected by the concentration of SP injected into virgin females (GLM with binomial data distribution: 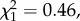 *p* = 0.50) nor by the interaction between concentration and population (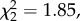 *p* = 0.40), but differed significantly between populations (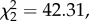 *p* < 0.001; electronic supplementary material, figure S5a). As we did not find an effect of concentration, we tested for an effect of SP receipt by comparing females injected with Ringer's solution with females injected with 10 pmol synthetic SP. Dahomey females reacted strongest to SP injection, with moderate and weak effects in Innisfail and Melbourne females, respectively (GLM with binomial data distribution: treatment × population: 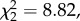 *p* = 0.012; electronic supplementary material, figure S5a).

By contrast, we found significant effects of both concentration (GLM with binomial data distribution: 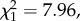 *p* = 0.005) and population (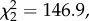 *p* < 0.001) but not their interaction (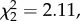 *p* = 0.348) on the proportion of females that mated 6 h after injection with synthetic SP (electronic supplementary material, figure S5b). Less females were willing to mate with increasing SP concentration injected in all three populations. Comparing 10 pmol SP versus Ringer's injected females showed that injection of SP overall decreased the proportion of females that mated (GLM with binomial data distribution: 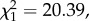 *p* < 0.001), with the extent significantly dependent on female population origin (treatment × population: 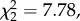 *p* = 0.02). Injection of 10 pmol SP strongly reduced female willingness to mate in Dahomey females but only weakly so in Innisfail and Melbourne females (electronic supplementary material, figure S5b).

### (d) SPR expression and SP/SPR haplotype networks

*SPR* was 1.04-fold higher expressed in the abdomen of Melbourne females compared to the other two populations (GLM with Gaussian data distribution: *F*_2*,*12_ = 7.65, *p* = 0.007; electronic supplementary material, figure S6).

**Figure 4.**
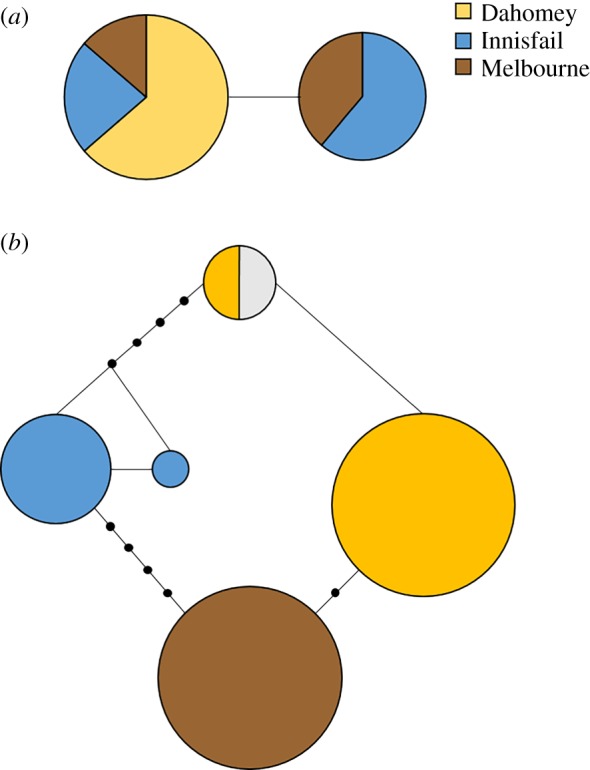
Haplotype networks of (*a*) the *SP* gene and (*b*) exon 6 of the *SPR* gene. Circle size represents the number of individuals carrying a specific haplotype and colour population affiliation. Each line segment represents a single mutational event with black dots indicating inferred haplotypes. Colours correspond to one of the three wild-type populations: yellow, Dahomey; blue, Innisfail; brown, Melbourne; grey, reference sequence from flybase (FBgn0029768).

The TCS haplotype network analysis of *SP* revealed two distinct haplotypes with only one mutational event separating them (figure 4*a*). Although all Dahomey samples shared the same haplotype, we detected a polymorphism for both haplotypes in the Australian populations. For exon 6 of *SPR*, we identified five distinct haplotypes with multiple events separating them with each population being characterized by its own distinct haplotype (figure 4*b*). Only the Melbourne population was made up entirely of one haplotype for this exon of *SPR*, while both Innisfail and Dahomey showed some low-level polymorphism composed of two different haplotypes separated by one mutational event.

## 4. Discussion

Our study shows for the first time significant variation in two female PMRs mediated by SP, egg-laying and remating behaviour [27], in three wild-type *D. melanogaster* populations and we hypothesized that SP and/or its receptor have diverged in these populations. While other SFPs might also be involved, SP and SPR have a major impact on these two PMRs [25,27]. In the next step, we disentangled male from female contributions by mating each sex separately to standardized mates. This revealed that males here did not differ in their ability to elicit female egg-laying and remating behaviour, suggesting that SP potency has not diverged. This was supported by the fact that genetic variability of the *SP* gene is low among these three populations. However, we found significant differences in female responses to SP indicating that females had diverged instead.

Our results reveal that Innisfail females are least responsive in terms of remating behaviour as they barely started to respond even to high doses of injected SP and, similarly, a single physiological dose from a single mating did not suppress remating as effectively as in Dahomey females. However, Innisfail females boosted their egg output by approximately 82% after receiving a physiological dose from a single mating (the two PMRs are regulated by independent pathways [47]). Interestingly, injecting synthetic SP at high doses did not induce an egg boost in Innisfail females. This could indicate that SP receipt alone is not enough to boost egg laying in these females and also the receipt of sperm and/or other SFPs are necessary. Alternatively, the concentration of synthetic SP used for injection might be simply too low to induce the egg boost (see below). While Melbourne females showed an intermediate phenotype, Dahomey females seemed most responsive. The latter react to both injection of synthetic SP and physiological receipt in single matings with increased egg-laying and suppressed remating behaviour and shortened lifespan when repeatedly receiving SP. Importantly, for females from all three populations, the effects of repeated SP receipt differently affected the estimate of LRS, female lifespan and the intrinsic rate of population increase *r*, which all serve as proxies of fitness, but not in the expected way.

Interestingly, we could not find any overall fitness costs of repeated SP receipt: SP was either beneficial (as in Innisfail and Melbourne) or neutral to fitness (Dahomey). This is in contrast to previous results also using Dahomey females, which showed a considerable cost [10]. One possible explanation is that females have overcome the cost of mating and evolved resistance to SP, i.e. females ‘won’ the sexual conflict at this particular time point and display no costs. Alternatively, the receipt of SP is only costly under certain conditions and otherwise beneficial to females. We will discuss both options in turn below.

Evolution of female resistance is a central part of sexually antagonistic coevolution theory [11] and is predicted to occur via two mechanisms [48–50]: either through shifts in (i) the female threshold value that elicits a PMR or (ii) female sensitivity to a male trait. According to theoretical models, the form of female resistance determines the dynamics of sexually antagonistic coevolution [48,50]. When sensitivity rather than the threshold evolves, then perpetual antagonistic changes are less likely [48] and females are predicted to suffer little fitness costs as we found here. By contrast, the evolution of the threshold leads to coevolutionary cycles [50]. However, both models highlight that the outcome depends on the strength of natural selection acting simultaneously and whether it constrains one form of resistance more than the other [48,50]. Empirical evidence for which of the two mechanisms is more likely to evolve is currently lacking. We tried to tackle this issue here by injecting females with different concentrations of synthetic SP to establish dose–response curves. A previous study [46] showed that SP elicited the PMR in a switch on/off manner indicating a threshold for SP, but here we needed much higher concentrations to effectively suppress remating or induce egg laying in the females. Importantly, for Innisfail and Melbourne females, we were unable to suppress remating or induce egg laying to the same extent as observed after a single mating (‘natural’ receipt of SP) even at the highest injected concentration (10 pmol). Remating suppression at least seemed to be concentration-dependent, in contrast to previous findings [46]. However, it is difficult here to judge conclusively whether resistance is modulated by a threshold or sensitivity as, particularly in Innisfail and Melbourne females, the SP concentrations we used did not induce a strong PMR, hence we might be well below their thresholds. Nonetheless, these females required a higher SP dose than e.g. Dahomey females, which supports the notion that females from these three populations differ in their responsiveness to SP, but sexually antagonistic coevolution is only one process that could explain the observed variation in female PMRs.

Alternatively, divergence in responsiveness to SP is not at all or only partially shaped by sexually antagonistic coevolution, and instead SP receipt is beneficial to females and intraspecific variation might be due to genetic drift or ecological variables. For example, the effect of SP is also dependent on female nutritional state [20] and shifts from antagonistic to beneficial. Hence, due to local adaptation, the underlying physiological trade-offs and/or metabolic pathways might be altered in these females potentially shifting life-history trade-offs as influenced by SP, and the effect on fitness across these populations. In addition, differences in population density and mate encounter rates might alter selection pressures as remating dynamics mediated through the SP receptor can shift reproductive interactions from expressing conflict to cooperation [31]. As the SPR also has another ligand (myoinhibiting peptides [51]) and is involved in other (unknown) functions, pleiotropic selection might have driven the observed sequence divergence in the SPR gene, which then altered the nature of male–female interactions and fitness effects of SP receipt. Hence, the expression of female mating costs might be condition-dependent and male–female coevolution as mediated by the SP–SPR molecular pair might be driven by neutral or positive selection under some conditions and shift towards an antagonistic interaction under others. Figuring out under which condition one or the other scenario occurs and how this affects male–female coevolutionary dynamics and population divergence needs further investigation.

Even though tentative, our genetic data for SPR support the phenotypic findings. We found that the three populations carry distinct haplotypes for exon 6 of the *SPR* suggesting that the observed phenotypic differences are rooted in the genetic variation found in *SPR.* Genetic variation in *SPR* and the interaction with *SP* have previously been found to affect male sperm defence success but not fertility or remating phenotypes [26].

We also found significant differences in *SPR* expression, but the difference was low (less than twofold change). Moreover, it is difficult to gauge to what extent this explains the phenotypic results, as intraspecific differences in *SP* and *SPR* expression are difficult to link to phenotypes [26]. However, one recent species comparison showed that overall high expression of *SPR* enhanced PMR responses [52]. Here, Melbourne females had a significantly higher *SPR* expression than Dahomey and Innisfail females, but they did not have the strongest response to SP receipt. This hints at *SPR* expression levels not correlating well with phenotypes, although testing more populations with known phenotypes like the DGRP isolines could provide further insights. Similarly, induction of egg laying and remating do not correlate linearly with *SP* expression in males [53,54]. Hence, expression levels might not explain phenotypic variation well and evolution at the *SPR* locus is more consistent with the divergence in female responsiveness to SP found here.

In conclusion, our study provides evidence for the evolution of female PMRs to receipt of SFPs such as SP. This opens up further research perspectives into male–female coevolution and whether SFPs are mediators of sexual conflict in general or only under specific conditions, taking into account the complexity of SFPs. In contrast to morphological traits that often have obvious fitness costs to females, SFPs can also be beneficial to females, e.g. through signalling the initiation of reproduction after receipt of sperm, and some SFPs need the cooperation of the female to be activated in the female reproductive tract after mating [4]. Previous ‘benefits to males and costs to females' schemes might simplify the complexity of these interactions too much. Another open question concerns the nature of female resistance traits: Do females modulate resistance via a threshold or sensitivity response? Hence, there is ample scope for future research to unravel the nature of female resistance that too often, at the level of physiological responses, is still a black box, and excitingly consider how these dynamics are influenced by ecology.

## Data accessibility

Phenotypic data and gene sequences are available from the Dryad Digital Repository: http://dx.doi.org/10.5061/dryad.c394c12 [55].
